# Age of First Exposure to Football Is Not Associated With Later-in-Life Cognitive or Mental Health Problems

**DOI:** 10.3389/fneur.2021.647314

**Published:** 2021-05-05

**Authors:** Grant L. Iverson, Jaclyn B. Caccese, Zachary C. Merz, Fionn Büttner, Douglas P. Terry

**Affiliations:** ^1^Department of Physical Medicine and Rehabilitation, Harvard Medical School, Boston, MA, United States; ^2^Spaulding Research Institute, Spaulding Rehabilitation Hospital, Charlestown, MA, United States; ^3^Sports Concussion Program, MassGeneral Hospital for Children, Boston, MA, United States; ^4^Home Base, A Red Sox Foundation and Massachusetts General Hospital Program, Charlestown, MA, United States; ^5^School of Health and Rehabilitation Sciences, The Ohio State University College of Medicine, Columbus, OH, United States; ^6^Department of Physical Medicine and Rehabilitation, University of North Carolina, UNC Memorial Hospital, Chapel Hill, NC, United States; ^7^LeBauer Department of Neurology, Moses H. Cone Memorial Hospital, Greensboro, NC, United States; ^8^Physiotherapy and Sports Science, School of Public Health, University College Dublin, Dublin, Ireland

**Keywords:** concussion, head trauma, traumatic brain injury, chronic traumatic encephalopathy, subconcussive

## Abstract

**Background:** The purpose of this study was to determine if earlier age of first exposure to football is associated with worse brain health in middle-aged and older adult men who played high school football.

**Methods:** Men from the United States, aged 35 and older, who reported playing high school football, completed a customized, online health survey via the Amazon Mechanical Turk (mTurk) platform. Survey items included physical, psychological, and cognitive symptoms over the past week and over the past year, sports participation history (including age of first exposure to football), medical history, and concussion history. Participants also completed the Patient Health Questionnaire-8 (PHQ-8) and the British Columbia Post-Concussion Symptom Inventory (BC-PSI).

**Results:** There were 186 men (age M = 51.78, SD = 10.93) who participated in high school football, and 87 (46.8%) reported football participation starting before the age of 12 and 99 (53.2%) reported football participation at or after the age of 12. Those who started playing football at an earlier age reported a greater number of lifetime concussions (M = 1.95, SD = 1.79) compared to those who started playing at age 12 or later (M = 1.28, SD = 1.52; U = 3,257.5, *p* = 0.003). A similar proportion of men who played football before vs. after the age of 12 reported a lifetime history of being prescribed medications for depression, anxiety, chronic pain, headaches, or memory problems. When comparing men who played football before vs. after the age of 12, the groups did not differ significantly in their ratings of depression, anger, anxiety, headaches, migraines, neck or back pain, chronic pain, concentration problems, or memory problems over the past week or the past year. The two groups did not differ significantly in their ratings of current symptoms of depression (PHQ-8; U = 4,187.0, *p* = 0.74) or post-concussion-like symptoms (BC-PSI; U = 3,944.0, *p* = 0.53). Furthermore, there were no statistically significant correlations between the age of first exposure to football, as a continuous variable, and PHQ-8 or BC-PSI scores.

**Conclusion:** This study adds to a rapidly growing body of literature suggesting that earlier age of first exposure to football is not associated with later-in-life brain health.

## Introduction

American football has been one of the most popular sports in the United States for decades, with more than one million youth participating at the high school level each year (1). In recent years, there have been growing public health concerns regarding the long-term effects of youth football participation. Among the general public, 54% of surveyed adults would not allow their child to play youth football (2). Among pediatricians queried, 77% would not allow their child to play youth football (3). Accordingly, over the past 10 years, there has been approximately a 10% decline in high school football participation (1), and several states, such as New Jersey, New York, Massachusetts, and Illinois, have held hearings or considered legislation to ban tackle football below the age of 12 (or before high school). Advocacy groups for this legislation have relied, in part, on some studies that have put forward a theory that playing football before the age of 12 is associated with psychiatric, cognitive, and neurological problems later in life (4–8). The origin of this theory appears to be a small study of former National Football League (NFL) players illustrating that those who started playing football before the age of 12, compared with those who started at age 12 or later, performed worse on a reading test and two neuropsychological tests (4). Follow-up studies by the same research group reported that earlier age of first exposure to football was associated with (i) differences in brain white matter microstructure in three sub-regions of the corpus callosum in former NFL players (5), (ii) smaller right thalamic volume in former NFL players (6), (iii) greater later-in-life behavioral and mood symptoms in former amateur and professional football players (7), and (iv) earlier age of cognitive and psychiatric symptom onset in deceased former amateur and professional football players (8).

With time, other research groups have independently conducted 11 studies using diverse samples and methodologies, with none observing an association between earlier age of first exposure to football (or other contact and collision sports) and worse clinical or brain imaging outcomes (9–19). In *current* high school and collegiate athletes, age of first exposure was not associated with objectively measured neurocognitive functioning, subjectively-experienced symptoms, or postural control across seven highly-powered, observational studies (12–16, 18, 19). Additionally, earlier age of first exposure to football was not associated with worse clinical outcome from concussion in a large study of *current* collegiate athletes (17). In a small clinical study of retired NFL players, there was no association between years of participation in pre-high school football and neurocognitive, neurological, or neuroimaging outcomes (9). A large survey study of more than 3,000 retired NFL players found no significant association between age of first exposure to football and increased risk of later-in-life anxiety, depression, or cognitive impairment (10). Finally, a recent online survey study of 123 men from the general population aged 35–55, who reported playing high school football, observed no significant differences between those starting before or after the age of 12 in the proportion of men who had been treated by a mental health professional, prescribed medications for mental health problems, or reported experiencing anxiety, depression, memory problems, chronic pain, or headaches over the past year (11).

The purpose of this study was to further examine if earlier age of first exposure to football was associated with worse brain health in middle-aged and older adult men who played high school football. The present study is a replication of a recently published study (11)—we include a larger sample size, extend the age range of the study sample to include men over the age of 55, and use more outcome variables. Given that (i) most studies to date have reported negative findings (9–20), (ii) some studies have reported positive findings but have also observed negative findings (4–8), and (iii) a recent small survey study of middle-aged men from the general population reported negative findings (11), we retained the null hypothesis that men who began playing football prior to the age of 12 would report similar symptoms of depression, anxiety, or cognitive problems compared with men who began playing football at age 12 or later.

## Materials and Methods

### Participants

Participants were recruited using Amazon Mechanical Turk (mTurk) (21), an online crowdsourcing platform used by social science and medical researchers (22) to efficiently collect large samples of survey participants (21). This platform has been used in studies relating to mental health and suicidality (23) and to recruit subjects with disabilities (24). We recruited men from the United States general population aged 35 and older. Only those who reported playing high school football were included in the analyses. There are no overlapping subjects in this survey compared with our prior survey (11). Differences between the present study and our prior study (11) include (i) a larger sample size, (ii) inclusion of men over the age of 55, (iii) self-reported diagnoses of medical and neurological problems (e.g., sleep apnea and Parkinson's disease; see Table 2), and (iv) symptom ratings from the past week (see Table 3).

### Measures

The Patient Health Questionnaire-8 (PHQ-8) is a brief, 8-item, self-report questionnaire that evaluates depression symptoms over the past 2 weeks. The PHQ-8 is a modified version of the PHQ-9 (25)–it excludes a single item that assesses thinking that you would be better off dead, or of hurting yourself. The PHQ-8 has been independently validated (26) and shown to be a useful evaluation of self-reported depression symptoms in the general public (27) and in primary care patient populations (28). Participants rate the frequency of symptoms and/or behaviors common to the experience of depression on a 0–3 scale. Higher scores represent greater symptoms. The cut-off score for screening positively for depression on PHQ-8 is 10 (26).

The British Columbia Post-Concussion Symptom Inventory (BC-PSI) (29, 30) is a 16-item self-report scale that assesses the frequency and severity of symptoms of post-concussion syndrome as defined in the 10th revision of the International Statistical Classification of Diseases and Related Health Problems (31). Participants rate the frequency and intensity of 13 concussion-related symptoms on a six-point scale (0–5), with higher values representing greater symptom frequency and intensity. Frequency and intensity ratings are multiplied together (i.e., how often x how bad) for each symptom to create a single symptom value. Single symptom values are then converted to item scores that represent both frequency and intensity (0–4). The 13-item scores are added to represent overall symptom burden. Three additional items assess the impact of symptoms on aspects of daily living, and they were not used in the current study.

Our study-specific survey assessed demographic information; physical symptoms (e.g., pain, headaches, migraines, sleep problems); psychological symptoms (e.g., depression, anger, anxiety); cognitive symptoms (e.g., memory, concentration, problem-solving); history of involvement in various sports; medical history; and concussion history. Each symptom was rated twice on a 1–5 scale (1 = never, 2 = rarely, 3 = sometimes, 4 = often, 5 = always) using the prompts “over the past week” and “over the past year.” We also relied on the PHQ-8 and the BC-PSI, described above, for current symptom reporting. Additionally, participants were asked if a medical provider ever diagnosed them with a variety of conditions, or if a provider recommended or prescribed medication for a variety of conditions. Participants were asked to estimate the number of concussions they had sustained, throughout their life, using the following definition as a guideline that was simultaneously displayed on the screen: *We define a concussion as a blow to the head or whiplash that caused any one or more of the following: (1) witnessed loss of consciousness (being “knocked out” and someone seeing it), (2) loss of memory for events immediately before and/or after the injury, or (3) feeling dazed and confused for at least 30 seconds*. Participants were also asked to estimate at what age and for how many years they spent participating in various sports, and for each sport, they provided an estimate of their participation duration (in years) in the following competition level categories: (i) before high school, (ii) during high school, (iii) during college, (iv) recreationally, (v), semi-professionally, and (vi) professionally. In an effort to reduce possible bias, participants were surveyed about their psychological health and cognitive functioning (i.e., completed the PHQ-8 and BC-PSI) before being asked questions about their sporting or concussion history.

### Procedures

Institutional review board approval was obtained prior to data collection. All data were collected via an online survey using the mTurk platform. Within this platform, mTurk “workers” are free to choose from a large variety of currently available tasks based on the name of the study or project, estimated completion time, and compensation rate. The title of the study was “Men's Health Survey” and the description read “Complete a survey about your health and concussion history.” Upon clicking, participants were provided an online informed consent document and compensation guidelines. They were informed that the study was designed to assess the prevalence of physical and mental health symptoms that may be associated with concussions or chronic traumatic encephalopathy in the general population. Participants could withdraw from the survey at any time.

Six attention-check validity items were embedded throughout the survey to assess for careless or random responding (i.e., “Choose [specific response] for the past week and [specific response] for the past year”). Only surveys in which all embedded validity items were answered satisfactorily were retained for data analysis. In addition, we searched for other possible indicators of careless responding. We identified a small number of respondents (*n* = 12; 5.4% of the sample) who either responded in a way inconsistent with the validity questions, endorsed having five or more the neurological diseases in our health history questionnaire, which seems extremely implausible (i.e., heart attack, stroke, Parkinson's Disease, arthritis, sleep apnea, amyotrophic lateral sclerosis, dementia, renal disease, cancer, or chronic traumatic encephalopathy), or completed the survey in <7 min. Regarding survey completion time, some surveys appeared to be completed so quickly that it is unlikely the participants read the items thoroughly. The survey completion time variable was non-normally distributed; we log-transformed this variable to normalize its distribution and excluded cases that were completed two or more standard deviations faster than the mean (which corresponded to a cutoff of 7 min). Some participants met more than one of those exclusion criteria. Financial compensation of $5.00 was provided.

### Statistical Analyses

Descriptive data were reported without adjustments for departures from normality or outliers. The sample was stratified into two groups: those who endorsed starting football prior to 12 years of age (*n* = 87) and those who reported starting football at age 12 or greater (*n* = 99). Selected demographic, BC-PSI, and PHQ-8 variables underwent visual inspection for normality, skewness, and kurtosis, as well as the Shapiro-Wilk tests of normality. Lifetime history of concussion, BC-PSI scores, and PHQ-8 scores were skewed. As such, non-parametric Mann-Whitney U analyses were conducted to assess for group-based differences amongst these variables. Chi-square analyses were used for nominal variables to examine group differences in the proportion who endorsed a specific health problem or condition. Finally, Spearman's Rho correlations were used to assess the association between age of first exposure (as a continuous variable), total years of football participation (as a continuous variable), and total scores on self-report measures. Significance was defined *a priori* as *p* < 0.05. All statistical analyses were performed with SPSS 26.0.

## Results

### Sample Characteristics

At total of 223 men who completed the survey endorsed playing high school football. Of those, 1 participant was excluded for answering embedded validity items incorrectly, 1 participant was excluded because he completed the survey too quickly (i.e., <7 min), and 10 participants were excluded because they gave implausible answers to a section of the survey involving neurological diagnoses in that they said “yes” to 5 or more diagnoses, leaving 211 participants. Of those, 24 reported playing semi-professional and/or professional football and were excluded to align with the inclusion criteria from our prior study and because our purpose was to study participation in amateur football. One additional man was excluded because he reported experiencing a concussion within the past year. The final sample included 186 men, 18 (9.7%) of whom also reported playing football in college. The survey was completed in a median of 18.5 min per participant [interquartile range (IQR) = 14.75–23.25, range = 8–46 min]. Their average age of first exposure to football was 11.42 years (SD = 2.98, Md = 12, IQR = 10–13.25). There were 87 (46.8%) men who reported football participation starting before the age of 12 (i.e., AFE <12) and 99 (53.2%) reporting football participation at or after the age of 12 (i.e., AFE ≥ 12). For men who started before age 12, their mean age of first exposure was 8.89 years (SD = 1.73, Md = 10, IQR = 8–10). For men who started at 12 years or older, their mean age of first exposure was 13.65 years (SD = 1.84, Md = 13, IQR = 12–15). Men with earlier exposure to football reported playing approximately twice as many years (M = 6.32, SD = 2.94) as those who started participation at age 12 or later (M = 3.64, SD = 2.26). The two groups did not differ significantly in their current age (t = −0.88, *p* = 0.38), time since most recent concussion (t = −0.10, *p* = 0.99), or body mass index (BMI; t = −0.61, *p* = 0.54). Sample characteristics are presented in Table 1.

**Table 1:** Demographic and Health Characteristics.

	**Total Sample**	**Started Football** ** <12 Years Old**	**Started Football** ** 12+ Years Old**
Age (M ± SD)	51.78 ± 10.93	51.02 ± 10.34	52.44 ± 11.44
**Education**			
Some High School	1 (0.5%)	0 (0%)	1 (1.0%)
High School Graduate/GED	15 (8.1%)	7 (8.0%)	8 (8.1%)
Some College/Technical Degree/AA	63 (33.9%)	32 (36.8%)	31 (31.3%)
College Degree (BA/BS)	69 (37.1%)	26 (29.9%)	43 (43.4%)
Advanced Degree (MA/MS, Ph.D., MD)	38 (20.4%)	22 (25.3%)	16 (16.2%)
**Income**			
Less than $30,000	37 (19.9%)	16 (18.4%)	21 (21.2%)
$30,000–$49,999	29 (15.6%)	15 (17.2%)	14 (14.1%)
$50,000–$79,999	52 (28.0%)	22 (25.3%)	30 (30.3%)
$80,000–$99,999	21 (11.3%)	11 (12.6%)	10 (10.1.%)
$100,000–$149,999	30 (16.1%)	18 (20.7%)	12 (12.1%)
More than $150,000	17 (9.1%)	5 (5.7%)	12 (12.1%)
Body Mass Index (M ± SD)	30.04 ± 7.77	29.67 ± 6.44	30.36 ± 8.79
Time Since Most Recent Concussion (years, M ± SD)	27.12 ± 12.96	27.11 ± 13.76	27.14 ± 14.31
Years Playing Football (years, M ± SD)	4.89 ± 2.92	6.32 ± 2.94	3.64 ± 2.26
**Race/Ethnicity (*****n*****, %)**			
White/Caucasian	159 (85.5%)	72 (82.8%)	87 (87.9%)
Black/African American	16 (8.6%)	10 (11.5%)	6 (6.1%)
Multiracial	5 (2.7%)	2 (2.3%)	3 (3.0%)
Asian/Asian-American	4 (2.2%)	1 (1.1%)	3 (3.0%)
Other	2 (1.1%)	2 (2.3%)	0 (0%)
Played College Football (*n*, %)	18 (9.7%)	8 (9.2%)	10 (10.1%)
**Marital Status (*****n*****, %)**			
Married	109 (58.6%)	53 (60.9%)	56 (56.6%)
Separated/Divorced	26 (14.0%)	12 (13.8%)	14 (14.1%)
Never Married	38 (20.4%)	17 (19.5%)	21 (21.2%)
Living with Partner	10 (5.4%)	4 (4.6%)	6 (6.1%)
Widowed	3 (1.6%)	1 (1.1%)	2 (2.0%)
**Exercise, Days Per Week (*****n*****, %)**			
0	17 (9.1%)	8 (9.2%)	9 (9.1%)
1–2	58 (31.2%)	29 (33.3%)	29 (29.3%)
3–4	57 (30.6%)	23 (26.4%)	34 (34.3%)
5–6	36 (19.4%)	19 (21.8%)	17 (17.2%)
7	18 (9.7%)	8 (9.2%)	10 (10.1%)
**Alcohol Use, Days Per Week (*****n*****, %)**			
0	87 (46.8%)	40 (46.0%)	47 (47.5%)
1–2	61 (32.8%)	24 (27.6%)	37 (37.4%)
3–4	20 (10.8%)	12 (13.8%)	8 (8.1%)
5–6	6 (3.2%)	5 (5.7%)	1 (1.0%)
7	12 (6.5%)	6 (6.9%)	6 (6.1%)
**Number of Alcoholic Beverages When Drinking (*****n*****, %)**			
I do not drink	71 (38.2%)	33 (37.9%)	38 (38.4%)
1	29 (15.6%)	13 (14.9%)	16 (16.2%)
2–3	55 (29.6%)	30 (34.5%)	25 (25.3%)
4–5	23 (12.4%)	9 (10.3%)	14 (14.1%)
6–8	3 (1.6%)	1 (1.1%)	2 (2.0%)
9+	5 (2.7%)	1 (1.1%)	4 (4.0%)
**Marijuana Use, Days Per Week (*****n*****, %)**			
0	158 (84.9%)	71 (81.6%)	87 (87.9%)
1–2	9 (4.8%)	7 (8.0%)	2 (2.0%)
3–4	6 (3.2%)	2 (2.3%)	4 (4.0%)
5–6	1 (0.5%)	1 (1.1%)	0 (0%)
7	12 (6.5%)	6 (6.9%)	6 (6.1%)
**Other Drug Use, Days Per Week (*****n*****, %)**			
0	179 (96.2%)	83 (95.4%)	96 (97.0%)
1–2	5 (2.7%)	2 (2.3%)	3 (3.0%)
3–4	2 (1.1%)	2 (2.3%)	0 (0%)
**Smoked 20 or more packs of cigarettes (*****n*****, %)**			
Yes, I currently smoke	33 (17.7%)	17 (19.5%)	16 (16.2%)
Yes, but I do not currently smoke	57 (30.6%)	24 (27.6%)	33 (33.3%)
No	96 (51.6%)	46 (52.9%)	50 (50.5%)
Lifetime History of Performance-Enhancing Drugs	12 (6.5%)	9 (10.3%)	3 (3.0%)

*Total Sample N = 186, <12 Years Sample n = 87, 12+ Years Sample n = 99. M, mean; SD, standard deviation, n, number of participants*.

### Lifetime Concussion History

Those who started playing football at an earlier age reported a greater number of lifetime concussions (M = 1.95, Md = 1, SD = 1.79, IQR = 1–3) compared with those who started playing at age 12 or later (M = 1.28, Md = 1, SD = 1.52, IQR = 0–2; Mann-Whitney U = 3,257.5, *p* = 0.003; Cliff's delta = 0.76). Those who started playing football before age 12 reported sustaining their first concussion at a younger age than those who started at age 12 or older (AFE <12: M = 16.91 years, SD = 10.17, Md = 15, IQR = 12–16; AFE≥12: M = 17.59 years, SD = 8.78, Md = 16, IQR = 14–18; Mann-Whitney U = 1,563.5, *p* = 0.025, Cliff's delta = 0.36).

### Health Problems

The proportion of men in both groups endorsing health conditions are presented in Table 2. There was a similar proportion of men in both groups who reported that they had been prescribed medications for depression, anxiety, chronic pain, headaches, or memory loss. There was no significant between-group difference in lifetime history of treatment by a mental health professional, although 42.5% of those who started played football prior to age 12 had gotten mental health treatment compared to 30.3% of those who started playing football at 12 or later. There was a similar proportion of men in both groups who reported being diagnosed with stroke, Parkinson's disease, dementia, arthritis, sleep apnea, heart attack, and cancer.

**Table 2:** Frequencies of health conditions across football exposure groups.

	**Total sample**	**Started Football** ** <12 Years Old**	**Started Football** ** 12+ Years Old**	**χ^2^**	***p***
**Medications for…**					
Attention-Deficit Hyperactivity Disorder	11 (5.9%)	3 (3.4%)	8 (8.1%)	1.79	0.18
Anxiety	38 (20.4%)	21 (24.1%)	17 (17.2%)	1.38	0.24
Depression	38 (20.4%)	16 (18.4%)	22 (22.2%)	0.42	0.52
Memory Loss	0 (0%)	0 (0%)	0 (0%)	–	–
Chronic Pain	108 (58.1%)	54 (62.1%)	54 (54.5%)	1.08	0.30
Headaches	34 (18.3%)	15 (17.2%)	19 (19.2%)	0.12	0.72
Diabetes	23 (12.4%)	12 (13.8%)	11 (11.1%)	0.31	0.58
High Cholesterol	56 (30.1%)	25 (28.7%)	31 (31.3%)	0.15	0.70
High Blood Pressure	81 (43.5%)	40 (46.0%)	41 (41.4%)	0.39	0.53
Congestive Heart Failure	8 (4.3%)	2 (2.3%)	6 (6.1%)	1.59	0.21
Arrhythmia/Atrial Fibrillation	8 (4.3%)	4 (4.6%)	4 (4.0%)	0.04	0.85
Liver Failure/Dysfunction	2 (1.1%)	2 (2.3%)	0 (0%)	2.30	0.13
Low Testosterone	10 (5.4%)	4 (4.6%)	6 (6.1%)	0.20	0.66
Erectile Dysfunction	25 (13.4%)	15 (17.2%)	10 (10.1%)	2.03	0.15
History of Seeing a Psychologist, Counselor, or Therapist	67 (36.0%)	37 (42.5%)	30 (30.3%)	3.00	0.08
**Diagnosed by a health care provider with…**					
Heart Attack	6 (3.2%)	2 (2.3%)	4 (4.0%)	0.45	0.50
Stroke	10 (5.4%)	4 (4.6%)	6 (6.1%)	0.20	0.66
Parkinson's Disease	2 (1.1%)	2 (2.3%)	0 (0%)	2.30	0.13
Arthritis	35 (18.8%)	19 (21.8%)	16 (16.2%)	0.98	0.32
Sleep Apnea	37 (19.9%)	20 (23.0%)	17 (17.2%)	0.98	0.32
Amyotrophic lateral sclerosis (ALS)	0 (0%)	0 (0%)	0 (0%)	–	–
Dementia	1 (0.5%)	1 (1.1%)	0 (0%)	1.14	0.29
Renal (kidney) disease	0 (0%)	0 (0%)	0 (0%)	–	–
Cancer	9 (4.8%)	4 (4.6%)	5 (5.1%)	0.02	0.89

*The first set of health problems were endorsed if the participant had a lifetime history of being recommended or prescribed medication for the condition. Total Sample N = 186, <12 Years Sample n = 87, 12+ Years Sample n = 99*.

A similar proportion of men in both groups endorsed significant problems with mental health, cognitive functioning, headaches, and chronic pain, over the past year and over the past week, are presented in Table 3. These rates are based on endorsing the symptom or problem as either “often” or “always.” There were no significant differences between groups in their ratings of depression, anger, anxiety, headaches, migraines, neck or back pain, chronic pain, concentration problems, or memory problems over the past week or the past year (all *p* > 0.05). A slightly, albeit non-significantly, higher proportion of those who started playing football prior to age 12 had migraines over the past year compared to those with started playing football at age 12 or later (7% vs. 2%; *p* = 0.06).

**Table 3:** Mental health, cognitive, and chronic pain problems in the past year and past week.

	**Total sample**	**Started Football** ** <12 Years Old**	**Started Football** ** 12+ Years Old**	**χ^2^**	***p***
**Past Year**					
Felt Depressed	28 (15.1%)	12 (13.8%)	16 (16.2%)	0.20	0.65
Anxiety	25 (13.4%)	10 (11.5%)	15 (15.2%)	0.53	0.47
Anger Problem	20 (10.8%)	10 (11.5%)	10 (10.1%)	0.09	0.76
Concentration Problems	24 (12.9%)	11 (12.6%)	13 (13.1%)	0.01	0.92
Memory Problems	16 (8.6%)	9 (10.3%)	7 (7.1%)	0.63	0.43
Headaches	16 (8.6%)	8 (9.2%)	8 (8.1%)	0.07	0.79
Migraines	9 (4.8%)	7 (7.0%)	2 (2.0%)	3.65	0.06
Chronic Pain	65 (34.9%)	32 (36.8%)	33 (33.3%)	0.24	0.62
Back or Neck Pain	52 (28.0%)	22 (25.3%)	30 (30.3%)	0.58	0.45
**Past Week**					
Felt Depressed	29 (15.6%)	13 (14.9%)	16 (16.2%)	0.05	0.82
Anxiety	25 (13.4%)	10 (11.5%)	15 (15.2%)	0.53	0.47
Anger Problem	20 (10.8%)	12 (13.8%)	8 (8.1%)	1.58	0.21
Concentration Problems	25 (13.4%)	13 (14.9%)	12 (12.1%)	0.32	0.57
Memory Problems	17 (9.1%)	8 (9.2%)	9 (9.1%)	0.00	0.98
Headaches	16 (8.6%)	7 (8.0%)	9 (9.1%)	0.06	0.80
Migraines	7 (3.8%)	5 (5.7%)	2 (2.0%)	1.78	0.18
Chronic Pain	65 (34.9%)	35 (40.2%)	30 (30.3%)	2.01	0.16
Back or Neck Pain	55 (29.6%)	30 (34.5%)	25 (25.3%)	1.89	0.17

*Total Sample N = 186, <12 Years Sample n = 87, 12+ Years Sample n = 99. The problems in this table were rated as “often” or “always” over the past year*.

### Current Symptoms

The two AFE groups did not differ significantly on current depressive symptoms (PHQ-8; U = 4,187.0, *p* = 0.74) or current concussion-like symptoms (BC-PSI; U = 3,944.0, *p* = 0.53; see Table 4). Similarly, the two AFE groups did not differ in the proportion of the sample who had a total PHQ-8 score of 10 or more (AFE <12 = 18.4%; AFE ≥ 12 = 24.2%; χ^2^ = 0.94, *p* = 0.33).

**Table 4:** PHQ-8 and BC-PSI total scores across football exposure groups.

	**Total Sample**				**Started Football** ** <12 Years Old**				**Started Football 12+** **Years Old**			
	**M**	**Md**	**SD**	**IQR**	**M**	**Md**	**SD**	**IQR**	**M**	**Md**	**SD**	**IQR**
PHQ-8 Total Score	5.09	4	5.14	1–8	5.15	4	5.13	1–8	5.03	3	5.18	1–9
BC-PSI Total Score	9.36	6	9.45	1–15	9.62	7	9.38	1.5–15	9.13	5	9.54	1–15.25

*Total Sample N = 186, <12 Years Sample N = 87, 12+ Years Sample N = 99. M, mean; Md, median; SD, standard deviation; IQR, interquartile range; PHQ-8, Patient Health Questionnaire-8; BC-PSI, British Columbia Post-Concussion Symptom Inventory (BC-PSI). Three participants (n = 3) had missing BC-PSI total scores*.

In the total sample, there was no significant correlation between age of first exposure to football, as a continuous variable, and PHQ-8 (Rho = 0.003, *p* = 0.97) or BC-PSI scores (Rho = −0.06, *p* = 0.41). In the subgroup who started playing football before age 12, there was no significant correlation between age of first exposure to football and PHQ-8 (Rho = −0.09, *p* = 0.41) or BC-PSI scores (Rho = −0.03, *p* = 0.76). Similarly, in the subgroup who started playing football at age 12 or older, there were no statistically significant correlations between age of exposure to football and PHQ-8 (Rho = 0.15, *p* = 0.13) or BC-PSI scores (Rho = −0.05, *p* = 0.60).

In the total sample, there was no significant correlation between number of years playing football and PHQ-8 (Rho = −0.12, *p* = 0.11) or BC-PSI scores (Rho = −0.05, *p* = 0.51). In the subgroup who started playing football before age 12, there was no significant correlation between number of years playing football and PHQ-8 (Rho = −0.12, *p* = 0.26) or BC-PSI scores (Rho = −0.13, *p* = 0.26). Similarly, the correlations between years playing football and PHQ-8 score (Rho = −0.18, *p* = 0.08) and BC-PSI scores (Rho = −0.06, *p* = 0.57) were non-significant in the group who started playing football at age 12 or older.

## Discussion

Considering that more than one million boys play high school football annually in the United States, it is reasonable to deduce that tens of millions of middle-aged and older adult men in the United States played high school football. For example, in a survey of 407 men between the ages of 35 and 55, nearly 1 in 3 (30.2%) reported participating in high school football^1^ ^1^. The present study replicated and extended our recently published study (11), which suggested that earlier age of first exposure to football was not associated with worse brain health in middle-aged men who played high school football. Similar to our previous findings, herein, playing football before the age of 12 was not associated with worse later-in-life brain health in middle-aged and older adult men from the general population. A similar proportion of men who played football before vs. after the age of 12 reported a lifetime history of being prescribed medications for depression, anxiety, chronic pain, headaches, or memory problems. When comparing men who played football before vs. after the age of 12, the groups did not differ significantly in their ratings of depression, anger, anxiety, headaches, migraines, neck or back pain, chronic pain, concentration problems, or memory problems over the past week or the past year. The two groups did not differ in their ratings of current symptoms of depression (PHQ-9) or post-concussion-like symptoms (BC-PSI), and there were no significant correlations between age of first exposure, as a continuous variable, or total years of exposure to football, and these two outcome measures. The men who started football before age 12 had significantly longer football careers, a greater number of lifetime concussions, and a younger age at first concussion compared with those who started playing football at 12 or older.

### Prior Studies With Former Football Players

In our previous survey study of 123 men between the ages of 35–55, similar proportions of both groups (i.e., age of first exposure <12 years vs. ≥12 years) reported a variety of health problems including depression, anxiety, anger, cognitive problems, headaches, migraines, or chronic pain (11). We replicated those specific findings in the present study. The current study is different from our previous study in that we (i) included men over the age of 55, (ii) examined self-reported diagnoses of other neurological conditions (e.g., Parkinson's disease, dementia, amyotrophic lateral sclerosis, stroke), and (iii) examined health outcomes experienced during the past week (in addition to over the past year, as was done in the prior study).

Other studies with retired NFL players have not found an association with age of first exposure. In a study of 45 former NFL players (mean age = 47), Solomon et al. (9) reported no association between years of pre-high school tackle football participation and current depressive symptoms, as well as MRI findings or neuropsychological test performance. In a very large cohort of 3,506 former NFL players, Roberts et al. (10) reported no association between age of first exposure to football and (i) depressive symptoms or problems with anxiety, (ii) being prescribed medications for anxiety or depression, or (iii) cognition-related quality of life.

Results from the aforementioned studies are inconsistent with, and did not replicate, any of the findings from the Boston University “Diagnosing and Evaluating Traumatic Encephalopathy using Clinical Tests (DETECT)” (4–6) and “Longitudinal Examination to Gather Evidence of Neurodegenerative Disease (LEGEND)” (7) studies suggesting that playing football before the age of 12 is associated with psychiatric, cognitive, and neurological problems later in life. It is important to note that the DETECT study recruited former NFL players (ages 40–69) with self-reported cognitive, behavioral, and mood symptoms for at least the last 6 months. Recruitment criteria for the LEGEND study were broader and included former high school, collegiate, and professional football players. In contrast to the findings in the DETECT study, Montenigro et al. (20) reported no significant association between earlier age of first exposure and cognition, behavioral, or mood symptoms among 93 former high school and collegiate LEGEND participants, but did find an association between symptom reporting and their “cumulative head impact index.” Thus, several studies to date in former football players have exclusively reported negative findings (9–11, 20) and some studies reporting positive findings have also observed negative findings (7, 8).

### Limitations

This study has several limitations. First, these survey results could have been influenced by reporting bias or inaccuracy. Some participants might have hastily completed the survey and responded carelessly. We tried to reduce that possible problem by excluding people based on internal validity checks (*n* = 1 excluded), completing the survey seemingly too quickly (*n* = 1), and by responding implausibly to having multiple neurological diseases (i.e., responding yes to 5 or more neurological disease questions; 10 men excluded; total excluded, *n* = 12, 5.4%). In addition, the online study description and consent form indicated that the purpose of the study was to assess the prevalence of chronic traumatic encephalopathy (CTE)-like symptoms and thus it is possible that some individuals who were concerned about their concussion history or who perceived themselves to be experiencing problems relating to past concussions (or sport participation) may have been more likely to participate. It is also possible that CTE awareness (from the media) could have influenced participants' beliefs about their personal risks for health problems, which in turn could have influenced some peoples' survey responses.

Second, by necessity, and consistent with all prior studies on this topic, age of first exposure was self-reported—and this methodology could be influenced by recall bias or simply be inaccurate. Third, the total years of playing football and the total number of lifetime concussions differed between the two groups, with those starting to play before the age of 12 having greater exposures on both of these variables. Theoretically, this should have increased the likelihood of finding group differences if one assumed that these exposure variables are related to long-term outcomes. Fourth, there was no way to verify the accuracy of their self-reported lifetime concussion history—a methodological limitation that is consistently present in this literature. Some individuals have difficulty recalling remote brain injuries that required *hospitalization* (32), so it is entirely possible that our results underestimate lifetime history of very mild concussions. Fifth, we did not control for multiple statistical comparisons by adjusting the *p*-value; however, the overall absence of statistically significant findings given this liberal significance threshold strengthens the likelihood that the null findings are indeed null. Sixth, more than 20% of our sample screened positively for depression, suggesting that those experiencing mental health difficulties were over-represented compared with men in the United States general population. The rate of screening positively for depression using the PHQ-8 in a random-digit-dialed phone survey was 8.6% (26). Other studies on this topic with former professional athletes have included subjects with much higher than expected rates of mental health difficulties (4, 7, 8). Finally, this study included only those men who were sufficiently cognitively intact to be part of this online survey platform. Only a very small percentage endorsed having a diagnosed neurological disease or condition.

## Conclusions

This study adds to a rapidly growing body of literature on this topic. Nine prior studies have considered clinical and neuroimaging outcomes in *former* amateur and professional football players (4–11, 20). Four (9–11, 20) of the nine studies reported no association between age of first exposure to football and later-in-life brain health problems. Moreover, in all studies of *current* high school and collegiate athletes, earlier age of first exposure to contact and collision sports has *not* been associated with worse neurocognitive functioning, subjectively-experienced symptoms, or postural control during preseason baseline testing (12–16, 18, 19), or worse clinical outcome following concussion (17). Considering the literature more broadly, separate from the issue of age of first exposure to football, results from 7 studies (33–38)^2^ have observed that men who played high school football are not at increased risk for later-in-life neurodegenerative disease (36, 37), and they do not report greater mental health problems in their 20s (33, 34), during middle age^2^, or during older adulthood (35, 38). In conclusion, the results of this study suggest that earlier age of first exposure to football is not associated with worse brain health in middle-aged and older adult men who played high school football.

## Data Availability Statement

Statistical analyses and outputs of all results used in this article will be made available by the authors, without undue reservation, to any qualified researcher.

## Ethics Statement

The studies involving human participants were reviewed and approved by University of North Carolina-Chapel Hill. The patients/participants provided their written informed consent to participate in this study.

## Author Contributions

GI conceptualized and designed the study, assisted with conducting the literature review, secured funding for the study, helped conceptualize the statistical analyses, wrote portions of the manuscript, edited drafts, and agrees to be accountable for the content of the work. JC assisted with conducting the literature review, edited drafts, and agrees to be accountable for the content of the work. ZM secured the IRB, programmed the survey, conducted the data collection, designed the database, edited drafts, and agrees to be accountable for the content of the work. FB assisted with conducting the literature review, edited drafts, and agrees to be accountable for the content of the work. DT helped conceptualize the statistical analyses, conducted the statistical analyses, wrote portions of the manuscript, edited drafts, and agrees to be accountable for the content of the work. All authors contributed to the article and approved the submitted version.

## Conflict of Interest

GI serves as a scientific advisor for NanoDX® (formerly BioDirection, Inc.), Sway Operations, LLC, and Highmark, Inc. He has a clinical and consulting practice in forensic neuropsychology, including expert testimony, involving individuals who have sustained mild TBIs (including athletes). He has received research funding from several test publishing companies, including ImPACT Applications, Inc., CNS Vital Signs, and Psychological Assessment Resources (PAR, Inc.). He has received research funding as a principal investigator from the National Football League, and subcontract grant funding as a collaborator from the Harvard Integrated Program to Protect and Improve the Health of National Football League Players Association Members. DT is a consultant for REACT Neuro, Inc. The remaining authors declare that the research was conducted in the absence of any commercial or financial relationships that could be construed as a potential conflict of interest.

## Footnotes

^1^Iverson GL, Merz ZC, Terry DP. High school football and midlife brain health problems. *Clin J Sport Med*. (in press).

^2^Iverson GL, Merz ZC, Terry DP. Playing high school football is not associated with increased risk for suicidality in early adulthood. *Clin J Sport Med*. (in press).
